# Supplementary figures and images for: Physical contact transmission of Cucumber green mottle mosaic virus by Myzus persicae
Source: PLoS One. 2021 Jun 23;16(6):e0252856. doi: 10.1371/journal.pone.0252856 (PMC8221510; doi:10.1371/journal.pone.0252856)

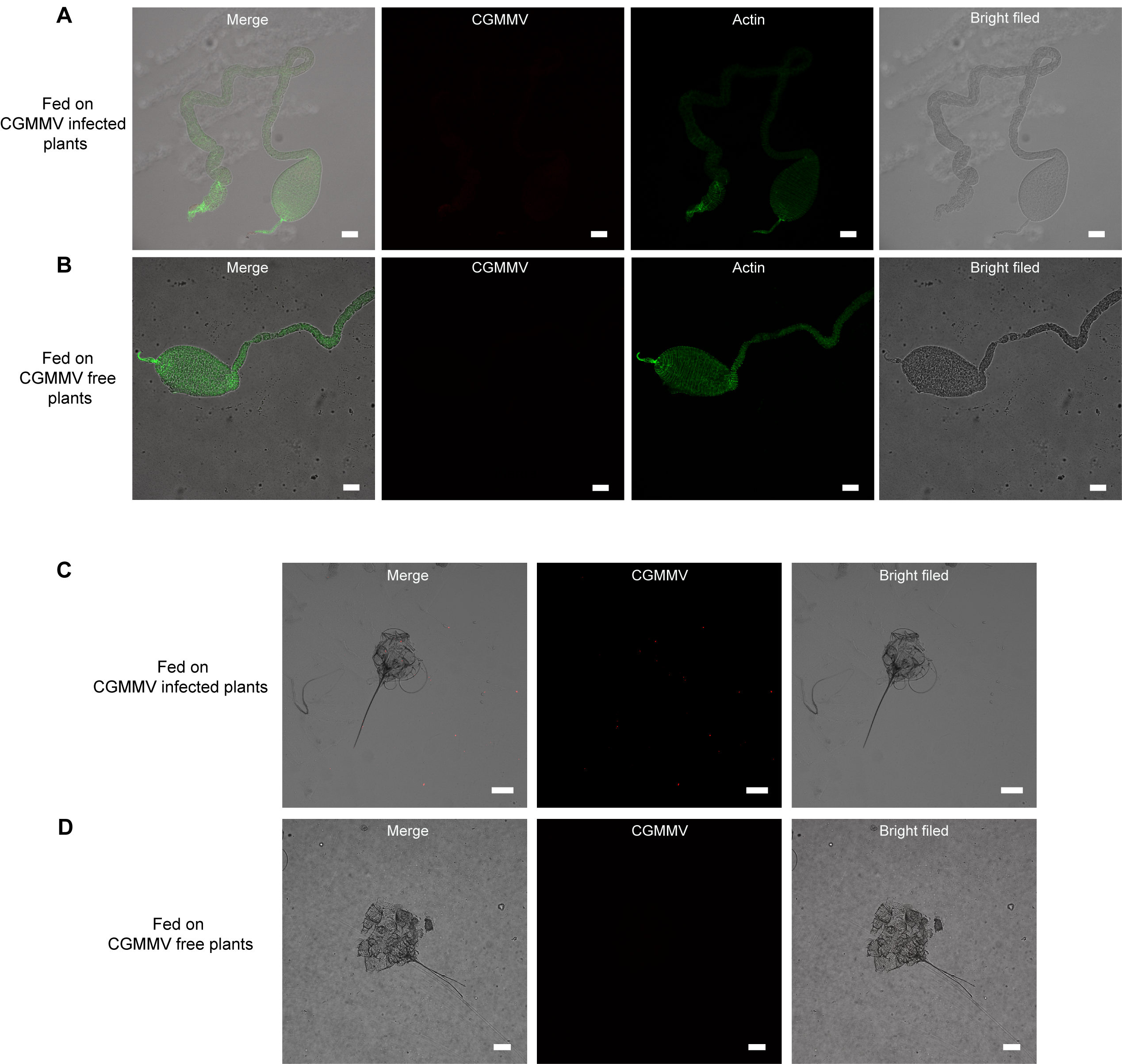

Supplement: S1 Fig — (A) Guts of M. persicae fed on CGMMV-infected plants. (B) Guts of M. persicae fed on CGMMV-free plants. (C) Stylets of M. persicae fed on CGMMV-infected plants. (D) Stylets of M. persicae fed on CGMMV-free plants. Red fluorescence signals indicate the localization of CGMMV in the guts or stylets of aphids. Green fluorescence signals indicate the actin gene in the guts of aphids. Scale bars, 100 μm. (TIF) [file pone.0252856.s001.tif]
