# Supplementary material for: Physical contact transmission of Cucumber green mottle mosaic virus by Myzus persicae
Source: PLoS One. 2021 Jun 23;16(6):e0252856. doi: 10.1371/journal.pone.0252856 (PMC8221510; doi:10.1371/journal.pone.0252856)
Supplement: S1 Table — (DOCX) [file pone.0252856.s002.docx]

**S1 Table. Primers used in this study**

| **Primer** | **Sequence (5' to 3')** |
| --- | --- |
| Aphid COI-F | ACAGGATGAACTATTTACCCACCC |
| Aphid COI-R | GCTCGTGTATCCACATCTATACCA |
| 28-CGMMV CP-F | CTGTATTTTCAGGGCCATATGGCTTACAATCCGATCACA |
| 28-CGMMV CP-R | ACGGAGCTCGAATTCGGATCCCTAAGCTTTC GAGGTGGTAG |
| CGMMV CP-F | ACAATCCGATCACACCTAGCA |
| CGMMV CP-R | CCGAAAACGCGGCTTCAAAT |
| UBC-F | TTTCGGTCCTGATGATACTCCC |
| UBC-R | CACAGAGCAAAGACTGGATTGA |
| Aphid Actin-F | GGTGTCTCACACACAGTGCC |
| Aphid Actin-R | CGGCGGTGGTGGTGAAGCTG |
