# Supplementary material for: Physical contact transmission of Cucumber green mottle mosaic virus by Myzus persicae
Source: PLoS One. 2021 Jun 23;16(6):e0252856. doi: 10.1371/journal.pone.0252856 (PMC8221510; doi:10.1371/journal.pone.0252856)
Supplement: S2 Table — (DOCX) [file pone.0252856.s003.docx]

**S2 Table. Transmission of CGMMV after viral acquisition through parafilm membrane by *M. persicae*.**

|  | **Parafilm-packaged leaves sap** | | | | **Parafilm-packaged leaves** |
| --- | --- | --- | --- | --- | --- |
| Different AAP^*^ (h) | 0.5 | 3 | 10 | 48 | 48 |
| Positive plants/Total plants (Percentage) | 0/15 (0%) | 0/10 (0%) | 0/10 (0%) | 0/10 (0%) | 0/12(0%) |

^*^AAP: Acquisition access period.
