# Supplementary material for: Physical contact transmission of Cucumber green mottle mosaic virus by Myzus persicae
Source: PLoS One. 2021 Jun 23;16(6):e0252856. doi: 10.1371/journal.pone.0252856 (PMC8221510; doi:10.1371/journal.pone.0252856)
Supplement: S3 Table — (DOCX) [file pone.0252856.s004.docx]

**S3 Table. Effect of CGMMV CP pre-treatment on virus acquisition of *M. persicae***

|  | **GFP pre-treatment** | **CGMMV CP pre-treatment** |
| --- | --- | --- |
| Positive aphids/Total aphids (Percentage) | 12/12 (100%) | 12/12 (100%) |
