# Supplementary material for: Physical contact transmission of Cucumber green mottle mosaic virus by Myzus persicae
Source: PLoS One. 2021 Jun 23;16(6):e0252856. doi: 10.1371/journal.pone.0252856 (PMC8221510; doi:10.1371/journal.pone.0252856)

Figure 1B

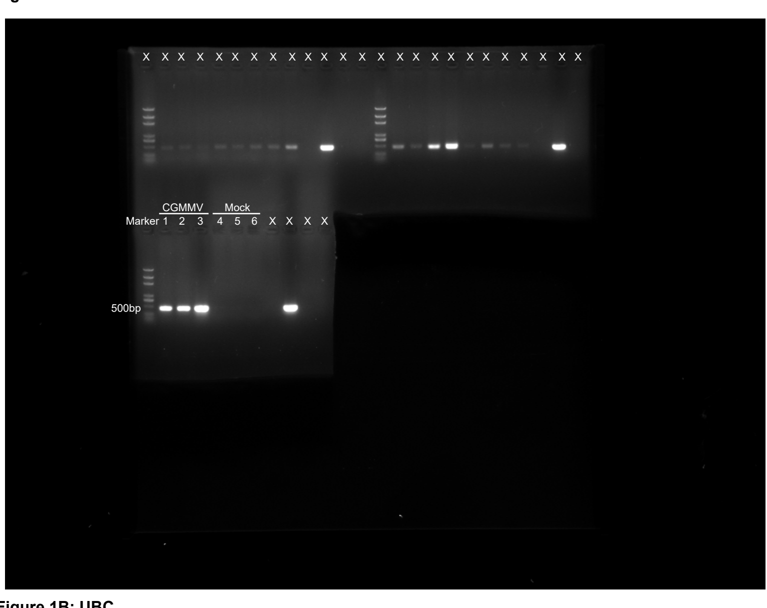

Figure 1B: UBC

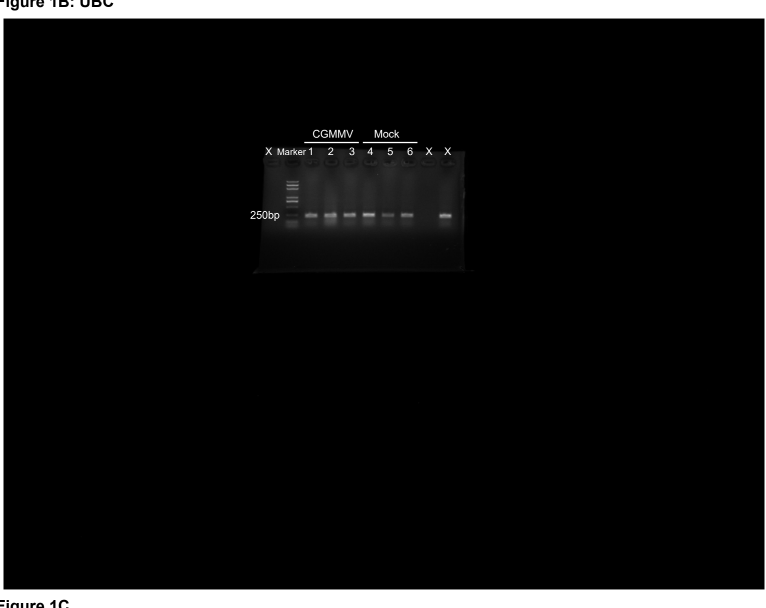

Figure 1C

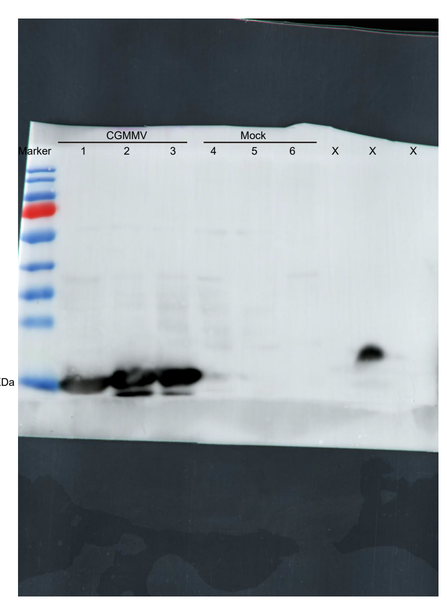

Figure 1C: UBC

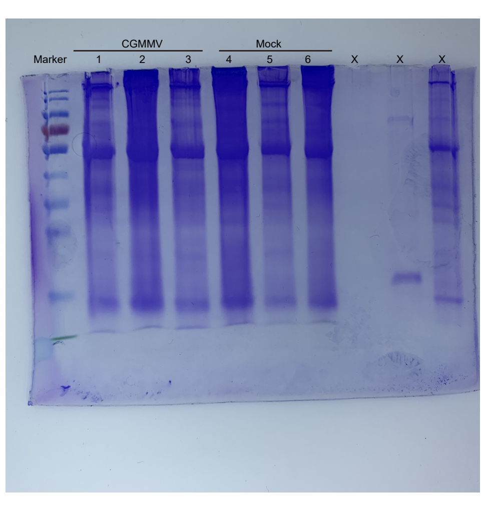

Figure 2

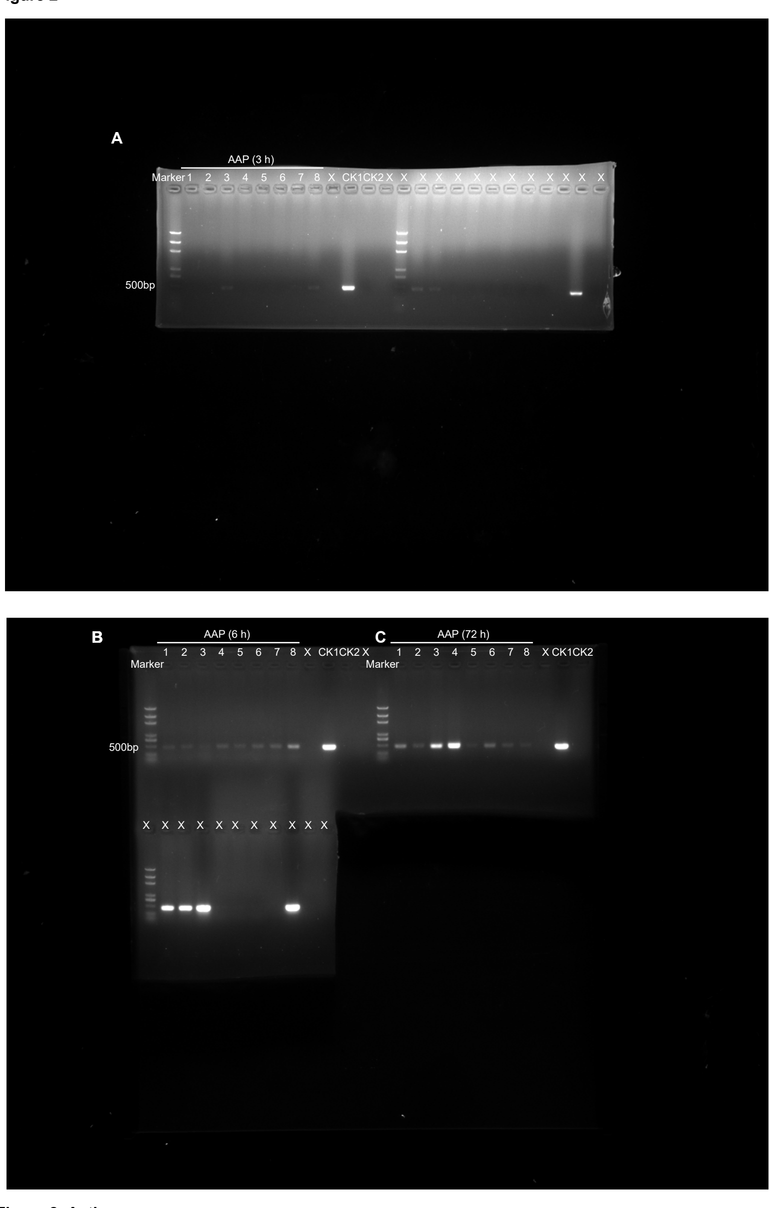

Figure 2: Actin

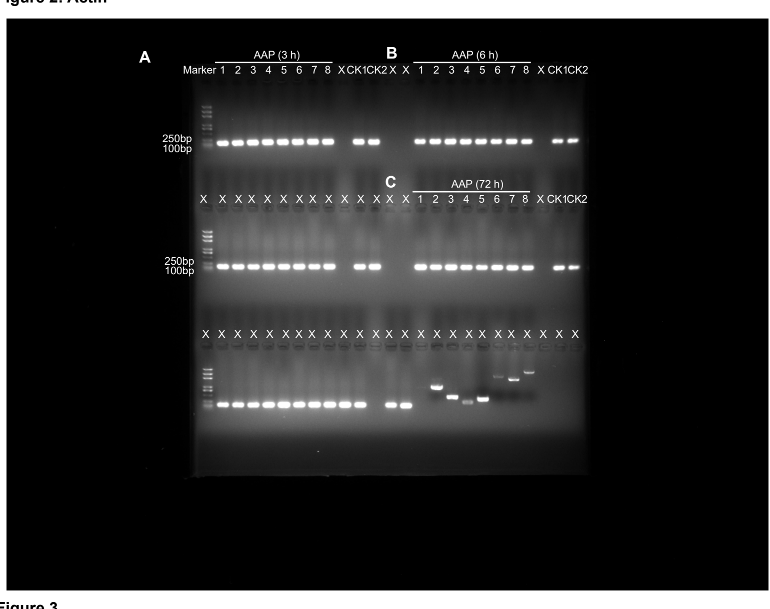

Figure 3

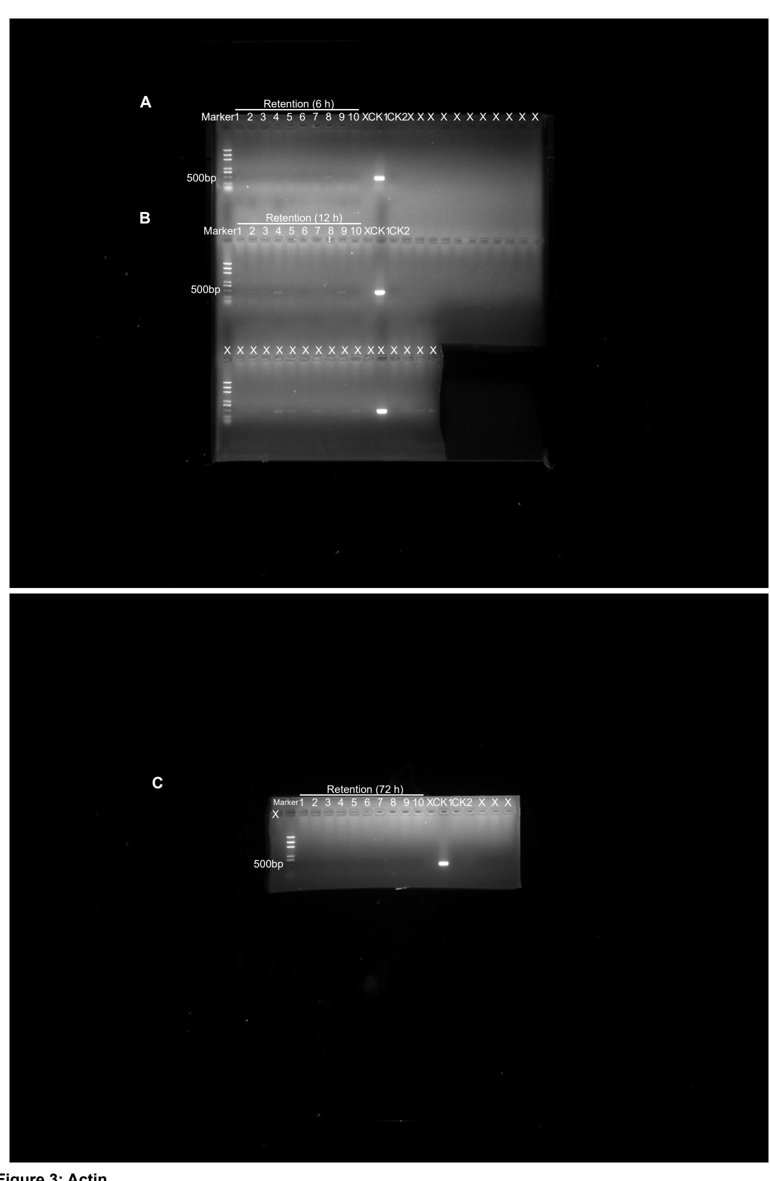

Figure 3: Actin

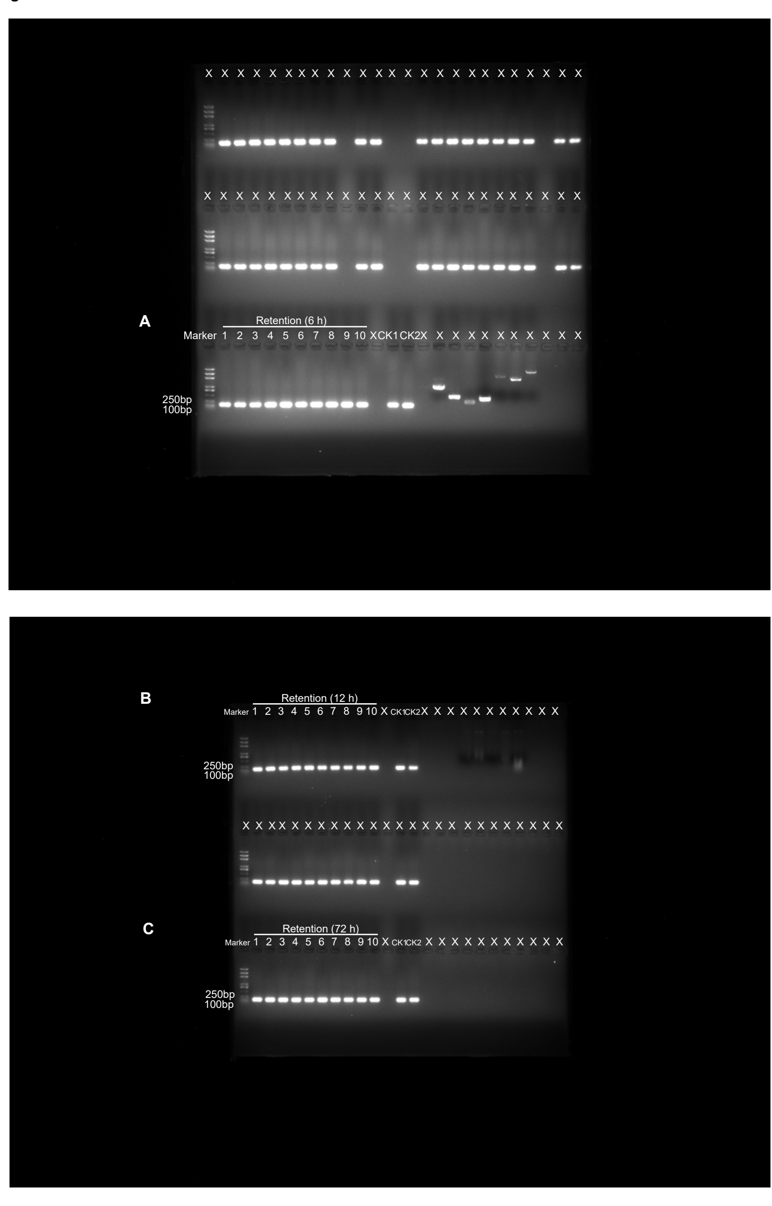

Figure 4B

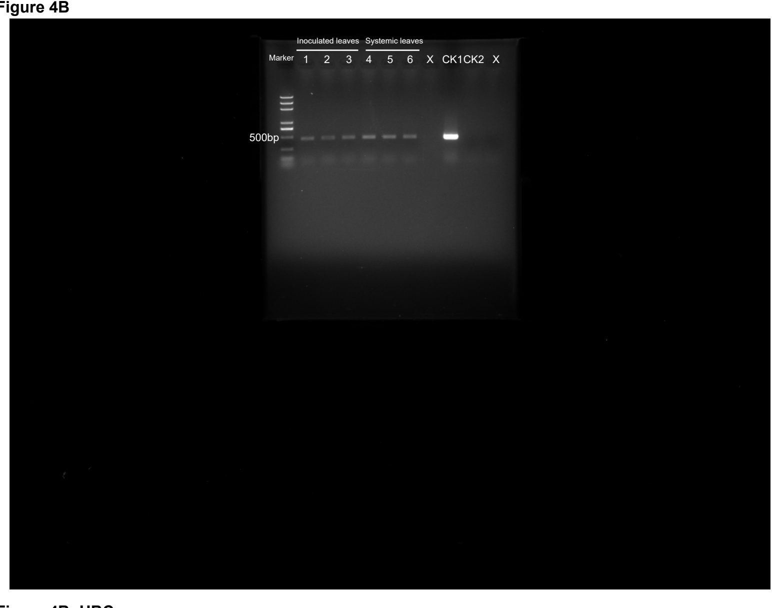

Figure 4B: UBC

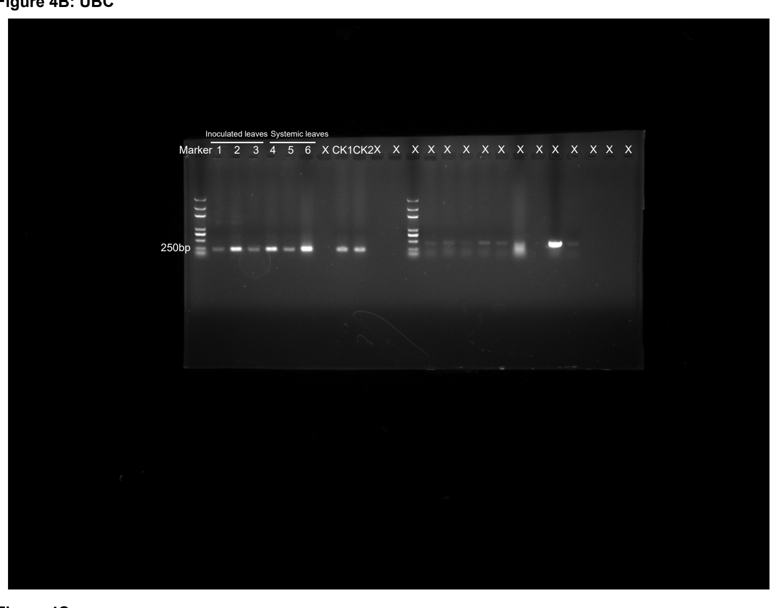

Figure 4C

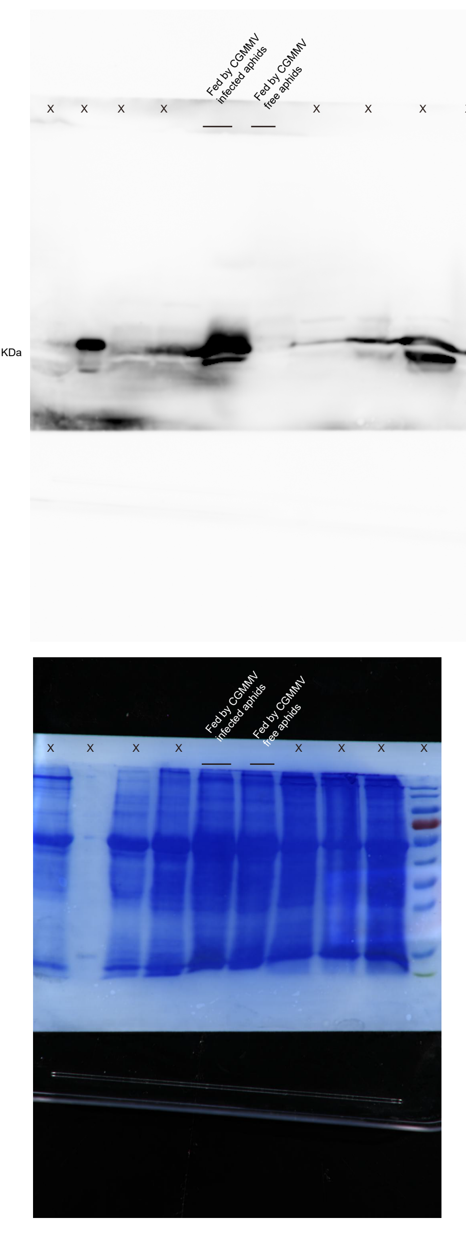

Supplement: S1 Raw images — Molecular weight markers were indicated in the left of each raw image, and each lane not included in the figure was marked with an “X” above the lane. (PDF) [file pone.0252856.s005.pdf]
