# Supplementary material for: Physical contact transmission of Cucumber green mottle mosaic virus by Myzus persicae
Source: PLoS One. 2021 Jun 23;16(6):e0252856. doi: 10.1371/journal.pone.0252856 (PMC8221510; doi:10.1371/journal.pone.0252856)
Supplement: Attachment — Submitted filename: Resonse to reviewers.docx [file pone.0252856.s006.docx]

**Comments to the Author**

1. Is the manuscript technically sound, and do the data support the conclusions?

The manuscript must describe a technically sound piece of scientific research with data that supports the conclusions. Experiments must have been conducted rigorously, with appropriate controls, replication, and sample sizes. The conclusions must be drawn appropriately based on the data presented.

Reviewer #1: Yes

Reviewer #2: Partly

**Response: We appreciate the valuable comments and suggestions for the two Reviewers. We have revised the manuscripts accordingly.**

2. Has the statistical analysis been performed appropriately and rigorously?

Reviewer #1: Yes

Reviewer #2: Yes

**Response: Thanks for the positive comments.**

3. Have the authors made all data underlying the findings in their manuscript fully available?

The [PLOS Data policy](http://www.plosone.org/static/policies.action#sharing) requires authors to make all data underlying the findings described in their manuscript fully available without restriction, with rare exception (please refer to the Data Availability Statement in the manuscript PDF file). The data should be provided as part of the manuscript or its supporting information, or deposited to a public repository. For example, in addition to summary statistics, the data points behind means, medians and variance measures should be available. If there are restrictions on publicly sharing data—e.g. participant privacy or use of data from a third party—those must be specified.

Reviewer #1: Yes

Reviewer #2: Yes

**Response: Thanks for the positive comments.**

4. Is the manuscript presented in an intelligible fashion and written in standard English?

PLOS ONE does not copyedit accepted manuscripts, so the language in submitted articles must be clear, correct, and unambiguous. Any typographical or grammatical errors should be corrected at revision, so please note any specific errors here.

Reviewer #1: No

Reviewer #2: Yes

**Response: The manuscript has been carefully revised by a native English speaker for improvement of English writing.**

5. Review Comments to the Author

Please use the space provided to explain your answers to the questions above. You may also include additional comments for the author, including concerns about dual publication, research ethics, or publication ethics. (Please upload your review as an attachment if it exceeds 20,000 characters)

Reviewer #1: The authors did lots of experiments to test the transmission of CGMMV by aphids. Though lots of data supported the idea that CGMMV can be transmitted by aphids, I donot agree the mechanical transimission by this insect. From these data, I suggest the authors to revise the paper to say that CGMMV can be transimitted by M. persicae by physical contaction. The aphids might get virions on the surface of their body.
**Response: Thank you very much for your valuable comments and we do agree with your suggestion. We have checked and replaced the word “mechanical” with** **“physical contact transmission” as suggested. Please see in the revised manuscript.**

L66. As more and more geminiviruses are found, the percent of aphid-borne viruses decreased.
The family names for animal and plants are not required in italic.
L74-79 Following the new writing style, the name of a virus is not in italic. Only describing the taxonomy of a virus species, the name must be italic.
**Response: Thank you for the suggestions. The change has been made in the revised manuscript (Please see Line68-85).**

How many cycles for RT-PCR in Figure 2 and 3? The bands for AAP and retension are faint. Usually, such faint bands are false positive amplification.
**Response: We are sorry for the confusion. Thirty cycles were set for the RT-PCR assay (Please see Line 173). To avoid false positives, we performed a negative control (CK2) that the aphids fed on CGMMV-free *N. benthamiana* plants in each experiment (Please see Figure 2 and 3).**

How many days it requires for mechanical transmission of CGMMV on N. benthamiana plants for systemic symptom development?
**Response: It takes 20 days for the systemic symptom post aphid fed (Please see revised Figure 4).**

Figure 4A.not CGMMV infected aphids. Western blot or ELISA is required to confirm the infection of CGMMV on aphid-mechnically transmitted plants.
**Response: Thank you for the suggestions. The Western blot assay has been added as suggested (Please see revised Figure 4).**

How to explain that successful CGMMV acquisition by M. persicae fed through the double-parafile membrane, but CGMMV was not detected in the stylets or midguts of M. persicae?
**Response: The first possibility might be that the low amount of CGMMV virion was hardly detected by immune-labeling. And another possibility is that virion was rinsed away during the experimental process because of the non-specific binding property (Please see Line398-400).**

L400-401 I donot agree that CGMMV can be successfully acquired by M. persicae. From the results in this study, I think that M. persicae contacts virions in low chance.

**Response: Thank you for your valuable comment and we do agree with your suggestion that *M. persicae* contacts virions in low chance.**

Reviewer #2: In this manuscript “Mechanical transmission of Cucumber green mottle mosaic virus by Myzuspersicae”, Qi et al. found that the M. persicae can acquire the CGMMV virions from virus-infected N. benthamiana and double-parafilm membrane containing leaf sap derived from the virus-infected N. benthamiana, and the virus can be retained by aphid for about 12 h after feeding on the N. benthamiana. Although the virus can be transmitted into healthy plant by aphids, the aphid feeding on the double-parafilm membrane cannot. In addition, the virus particles were not detected in the aphids. These results suggest that the M. persicae transmitted the virus by mechanical contact.
**Response: Thank you very much for your valuable comments.**

Many references in the introduction section are out of date. For example, Line 56-58, there are several other tissues have been identified as the initial infection site for different viruses, not only the midgut. I suggest that new research progresses should be added.
**Response: We do agree with your suggestion, and new references are added (Please see Line 57 in the revised manuscript).**

Line 35, 256, “CGGMV” should be “CGMMV”.
**Response: We are sorry for the mistake. We have revised it (Please see Line 35, 257 in the revised manuscript.**

Figure 1A, the growth stage of the two plant are obviously different. Mock plant seems not to be agroinfiltrated and much younger than the left N. benthamiana plant.

**Response: We do agree with your comments, and mock plant is replaced (Please see Figure 1A)**.

6. PLOS authors have the option to publish the peer review history of their article ([what does this mean?](https://journals.plos.org/plosone/s/editorial-and-peer-review-process#loc-peer-review-history)). If published, this will include your full peer review and any attached files.


If you choose “no”, your identity will remain anonymous but your review may still be made public.


**Do you want your identity to be public for this peer review?** For information about this choice, including consent withdrawal, please see our [Privacy Policy](https://www.plos.org/privacy-policy).

Reviewer #1: No

Reviewer #2: No

**Response: Thanks for the positive comments.**
